# Interfinger Differences in Pulse Oximetry Signal-Derived Peripheral Perfusion Index: A Single-Center Exploratory Study

**DOI:** 10.7759/cureus.82131

**Published:** 2025-04-12

**Authors:** Ryosuke Shintani, Motohiro Sekino, Shuntaro Sato, Takayuki Morimoto, Shohei Kaneko, Naoya Iwasaki, Hiroshi Araki, Taiga Ichinomiya, Ushio Higashijima, Tetsuya Hara

**Affiliations:** 1 Department of Anesthesiology and Intensive Care Medicine, Nagasaki University Graduate School of Biomedical Sciences, Nagasaki, JPN; 2 Clinical Research Center, Nagasaki University Hospital, Nagasaki, JPN

**Keywords:** abnormal peripheral perfusion, perfusion index, peripheral perfusion index, peripheral perfusion monitoring, pulse amplitude index

## Abstract

Introduction: Peripheral perfusion monitoring is crucial for the management of critically ill patients because abnormal peripheral perfusion is associated with a poor prognosis. The peripheral perfusion index (PPI), derived from pulse oximetry, quantifies peripheral perfusion but varies across fingers. A pulse oximeter probe may cause burns when worn at the same site. Therefore, changing the site has been recommended. However, changes in PPI values owing to probe replacement reduce the reliability of clinical and research applications. No two fingers with equivalent PPI values have been identified yet. This study assessed the interfinger differences in PPI by measuring the five fingers simultaneously and identified the two fingers with the least fluctuation in values.

Methods: A total of 30 healthy volunteers were included in this single-center prospective exploratory study. For PPI measurements, the pulse amplitude index (PAI) was measured using a pulse oximeter on a bedside monitor (Life Scope PT; Nihon Kohden Corp., Tokyo, Japan). The tape-type disposable pulse oximeter probes were attached to each of the five fingertips of the dominant hand, and measurements were conducted in three rooms with different temperatures (16°C, 22°C, and 28°C) to induce thermoregulatory responses and capture a wide range of PAI values. The primary endpoint was the PAI in pairs of two fingers each (a total of 10 pairs). Paired t-tests with Bonferroni correction were used to compare finger pairs, with statistical significance defined as *p* < 0.005.

Results: The combination with the least difference in PAI was the index-ring finger combination (0.07% ± 1.89%, *p* = 0.74), followed by the middle-ring finger (0.45% ± 1.93%, *p* = 0.03) and index-middle finger combinations (0.52% ± 2.05%, *p* = 0.02). All other finger combinations showed statistically significant differences (*p* < 0.001).

Conclusion: PPI values measured using the pulse oximeter showed the smallest interfinger difference between the index and ring fingers. If changing the finger to which the probe is attached during clinical or research use is needed, it may be possible to consistently measure the PPI values by alternately attaching the probe to the index and ring fingers.

## Introduction

Abnormal peripheral perfusion or peripheral hypoperfusion has been reported to be associated with poor prognosis in patients with septic shock [1,2], cardiogenic shock [3], and high-risk surgery [4]. A recent study conducted in patients with septic shock found that initial resuscitation aimed at improving abnormal peripheral perfusion, as assessed by capillary refill time, had a better prognosis than resuscitation aimed at improving lactate levels [5,6]. Additionally, “perfusion-centered” approaches, which focus on peripheral perfusion as an essential indicator of organ perfusion, are expected to be more effective for improving prognosis than “pressure-guided” strategies focused on maintaining mean blood pressure [7,8]. Consequently, peripheral perfusion monitoring is crucial in the management of critically ill patients.

The pulse oximeter, a widely used monitor in clinical practice, not only evaluates peripheral oxygen saturation but also presents a pulse waveform that reflects peripheral tissue perfusion [9]. This is quantified as the peripheral perfusion index (PPI) and calculated as the ratio of the pulsatile component to the nonpulsatile component of the transmitted infrared light intensity [10,11]. In contrast to capillary refill time, PPI can quantitatively and continuously evaluate peripheral perfusion; therefore, they have been used in many clinical studies in recent years [9,12].

Pulse oximeter probes are usually attached to the index or middle fingers [13]; however, the PPI values vary between fingers [14,15]. A pulse oximeter probe may cause burns when worn at the same site for a long time, especially in patients with peripheral hypoperfusion [16,17]; therefore, changing the site frequently has been recommended [18]. However, changes in PPI values owing to probe replacement reduce the reliability of clinical and research applications. Previous studies that reported the interfinger differences in PPI values have the problem that they evaluated by sequentially changing one probe on each finger [14,15]. As peripheral perfusion fluctuates from moment to moment in response to neural activity and other factors [19], these studies may not have been able to evaluate PPI at the same point in time. Additionally, when a clip-type probe was used [15], differences in finger thickness may have caused changes in the pressure exerted on the finger tissue, which affected the PPI value [20]. Overall, no two fingers with equivalent PPI values suitable for probe replacement have yet been identified.

The purpose of this study was to verify the interfinger differences in PPI by measuring the five fingers simultaneously using tape-type probes and to identify the two fingers that have the least fluctuation in values.

## Materials and methods

Study design

This single-center prospective exploratory study was conducted in the operating room of Nagasaki University Hospital (Nagasaki, Japan). This study was approved by the Ethics Committee for Medical Sciences, Nagasaki University Graduate School of Biomedical Sciences (no. 23022401) on March 16, 2023, and followed the principles of the Declaration of Helsinki. Written informed consent was obtained from each participant before commencing the study. This study adhered to the Strengthening the Reporting of Observational Studies in Epidemiology guidelines.

Participant selection

The study recruitment period was from April 1, 2023, to June 30, 2023. The participants were healthy adult volunteers. Individuals with vascular diseases, finger pigment disorders, or nail decorations were excluded.

Study protocol and measurements

**Figure 1:**
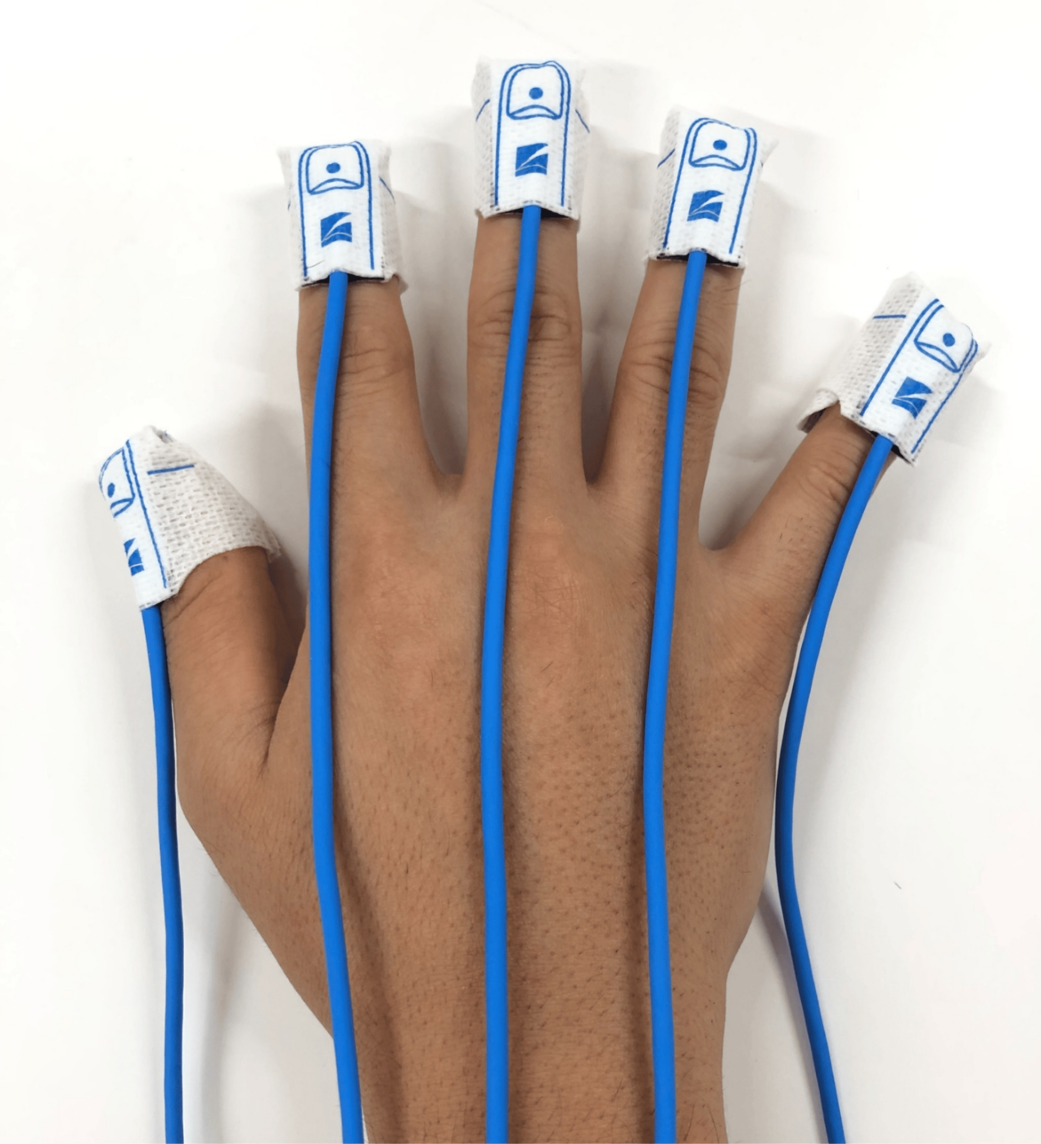
Tape-type pulse oximeter probes attached to each of the five fingers of the dominant hand

For PPI measurements, the pulse amplitude index (PAI) was measured using a pulse oximeter on a bedside monitor (Life Scope PT; Nihon Kohden Corp., Tokyo, Japan). The PAI was calculated as the ratio of the pulsatile component to the non-pulsatile component of the transmitted infrared light intensity using the same principle as the perfusion index (Masimo Corp., Irvine, CA, USA) [9,10,21]. To maintain constant measurement conditions, tape-type disposable pulse oximeter probes (TL-271T; Nihon Kohden Corp.) were attached to all five fingertips of the dominant hand, based on findings from previous studies (Figure 1) [14,15]. To accurately measure the PAI at the attachment site, the probe was attached to the fingertip such that it was closely fitted to the fingertip without exerting pressure. Additionally, non-transparent black gloves were used to prevent interference from the transmitted light between adjacent probes and room light.

All measurements of the PAI and attaching probes were performed in the operating room by a specific clinician. Measurements were conducted in three temperature-controlled rooms (16°C, 22°C, and 28°C) to induce thermoregulatory responses [20,22] and capture a wide range of PAI values. Each participant was assessed in all three rooms in a randomized order. Simple block randomization was used to ensure a balanced allocation of participants across all temperature sequences. Before measurements in each room, participants rested in the supine position for 5 minutes.

Peripheral oxygen saturation, pulse rate, mean blood pressure, and body temperature were also measured. The thickness of each finger was measured on the nail bed using calipers. The visual analog scale was used to measure the coldness or hotness that the participants felt in each room, with lower values defined as colder.

Study endpoints

The primary endpoint of this study was the PAI in pairs of two fingers each (a total of 10 pairs). The secondary endpoint was fingertip thickness in pairs of two fingers each (a total of 10 pairs), the correlation between fingertip thickness and PAI (averaged values obtained at 16°C, 22°C, and 28°C), and the correlation between the mean PAI of five fingers and the difference between the maximum and minimum PAI at 16°C, 22°C, and 28°C.

Statistical analyses

The study design focused on the feasibility of the study at our institution, and no statistical sample size calculations were conducted. Data are presented as means ± standard deviation for quantitative variables and as counts and percentages for categorical variables. The paired t-test was used for comparisons of PAI in pairs of two fingers each (a total of 10 pairs), and similarly, it was used to compare fingertip thickness between these finger pairs. To assess the impact of fingertip thickness on PAI values, Pearson’s correlation coefficient (r) was used to examine the correlation between fingertip thickness and PAI. To clarify the effect of variations in PAI values on interfinger differences, the same method was used to examine the correlation between the mean PAI of the five fingers and the difference between the maximum and minimum PAI. All tests were two-sided. For comparisons conducted using the paired t-test, the Bonferroni correction was applied, with statistical significance set at *p* < 0.005. For Pearson's correlation analysis, statistical significance was defined as *p* < 0.05. However, as the sample size was not statistically calculated and no correction for multiplicity was performed, the statistical significance should be interpreted only as exploratory. Statistical analyses were performed using the JMP Pro 14 software (SAS Institute Inc., Cary, NC, USA).

## Results

Study population

A total of 30 healthy volunteers were included in this study. Participants’ characteristics are presented in Table 1. The mean participant age was 31 years, and 40% of the participants were male. Additionally, 93% of the participants were right-handed. Table 2 shows descriptive statistics of the data obtained at 16°C, 22°C, and 28°C and at all room temperatures combined. A wide range of PAI data was collected owing to the thermoregulatory response.

**Table 1:** Baseline characteristics of the study population Values are presented as mean ± standard deviation or number of participants (%).

	Overall (n = 30)
Age (years)	31 ± 5
Sex: male	12 (40)
Dominant hand: right	28 (93)
Body temperature (℃)	36.7 ± 0.3
Thickness of fingertip (mm)	
Thumb	12.50 ± 1.56
Index finger	10.71 ± 1.17
Middle finger	11.56 ± 1.05
Ring finger	10.38 ± 0.95
Little finger	9.47 ± 1.08

**Table 2:** Measurements at each room temperature Values are presented as mean ± standard deviation. The visual analog scale evaluates the degree of coldness or hotness of a participant in relation to room temperature on a scale of 1 to 10; the lower the number, the colder the participant.

	16℃ (n = 30)	22℃ (n = 30)	28℃ (n = 30)	Overall (n = 90)
Pulse amplitude index (%)				
Thumb	2.93 ± 1.94	4.49 ± 2.13	5.96 ± 2.36	4.46 ± 2.48
Index finger	4.14 ± 2.68	6.02 ± 2.82	7.53 ± 2.64	5.90 ± 3.05
Middle finger	4.53 ± 3.01	6.52 ± 3.41	8.22 ± 3.45	6.42 ± 3.62
Ring finger	4.43 ± 3.20	6.12 ± 3.16	7.35 ± 2.64	5.97 ± 3.24
Little finger	3.59 ± 2.75	5.49 ± 2.92	6.67 ± 2.91	5.25 ± 3.13
Peripheral oxygen saturation (%)				
Thumb	99 ± 1	98 ± 1	98 ± 1	98 ± 1
Index finger	99 ± 1	98 ± 1	98 ± 1	98 ± 1
Middle finger	99 ± 1	99 ± 1	99 ± 1	99 ± 1
Ring finger	99 ± 1	99 ± 1	99 ± 1	99 ± 1
Little finger	99 ± 1	99 ± 1	99 ± 1	99 ± 1
Mean blood pressure (mmHg)	89 ± 9	87 ± 8	86 ± 7	87 ± 7
Pulse rate (beats/min)	68 ± 8	69 ± 9	70 ± 10	69 ± 9
Visual analog scale	3 ± 1	5 ± 1	7 ± 1	5 ± 2

Primary endpoint

**Figure 2:**
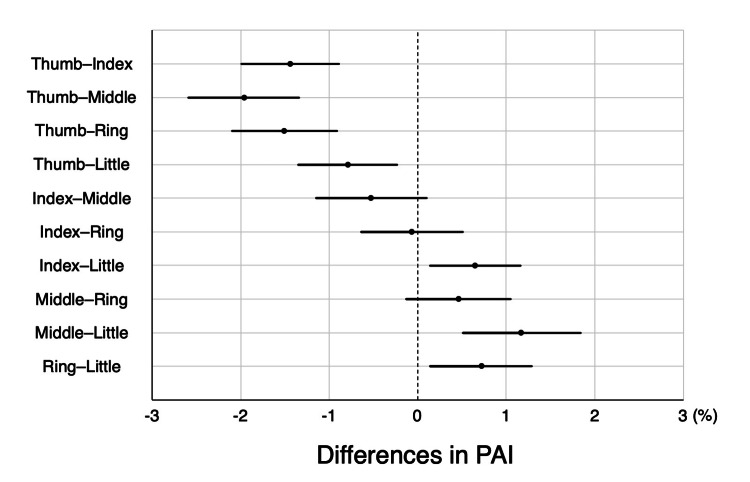
Comparison of PAI in pairs of two fingers each The figure shows the mean and 99.5% confidence interval of the difference in PAI in pairs of two fingers each. The combination with the least difference in PAI was the index-ring finger combination (0.07% ± 1.89%, *p* = 0.74), followed by the middle-ring finger (0.45% ± 1.93%, *p* = 0.03) and index-middle finger combinations (0.52% ± 2.05%, *p* = 0.02). PAI: pulse amplitude index

The combination with the least difference in PAI was the index-ring finger combination (0.07% ± 1.89%, *p* = 0.74), followed by the middle-ring finger (0.45% ± 1.93%, *p* = 0.03) and index-middle finger combinations (0.52% ± 2.05%, *p* = 0.02) (Figure 2). All other finger combinations showed statistically significant differences (*p* < 0.001).

Secondary endpoint

**Figure 3:**
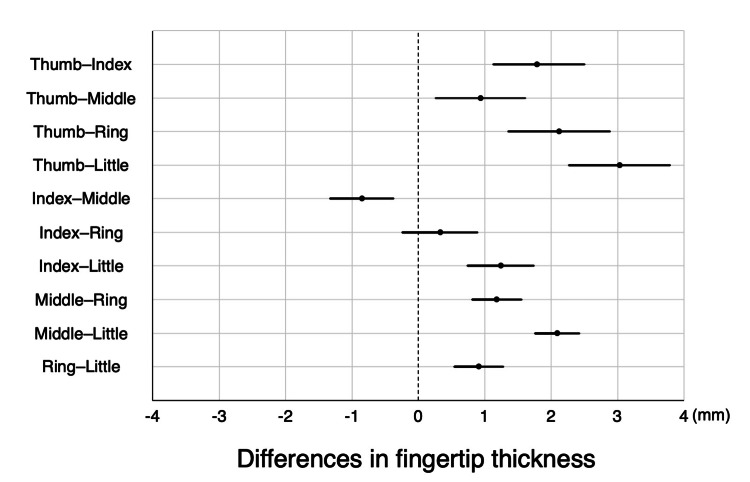
Comparison of fingertip thickness in pairs of two fingers each The figure shows the mean and 99.5% confidence interval of the difference in fingertip thickness in pairs of two fingers each. The combination with the least difference in fingertip thickness was the index-ring finger combination (0.33 ± 1.00 mm, *p* = 0.09), followed by the index-middle finger (0.85 ± 0.84 mm, *p* < 0.001) and ring-little finger combinations (0.91 ± 0.66 mm, *p* < 0.001).

**Figure 4:**
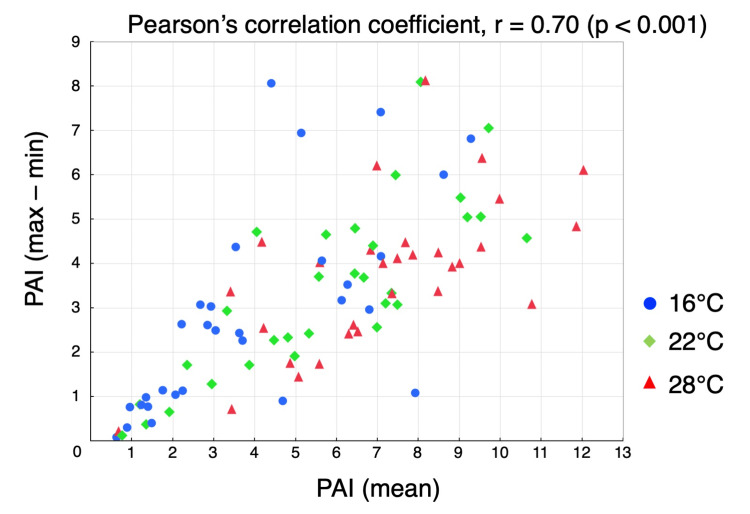
Correlation between the mean PAI of the five fingers and the difference between the maximum and minimum PAI values Blue circles, green diamonds, and red triangles indicate data at room temperatures of 16°C, 22°C, and 28°C, respectively. There was a relatively high correlation between the mean PAI of the five fingers and the difference between the maximum and minimum PAI values (r = 0.70, *p* < 0.001). PAI: pulse amplitude index

The combination with the least difference in fingertip thickness was the index-ring finger combination (0.33 ± 1.00 mm, *p* = 0.09), followed by the index-middle finger (0.85 ± 0.84 mm, *p* < 0.001) and ring-little finger combinations (0.91 ± 0.66 mm, *p* < 0.001) (Figure 3). No correlation was observed between fingertip thickness and PAI (r = 0.06, *p* = 0.49). However, a relatively strong positive correlation was observed between the mean PAI of the five fingers and the difference between the maximum and minimum PAI values (r = 0.70, *p* < 0.001) (Figure 4).

## Discussion

The PPI calculated from pulse oximeters, such as the PAI, is a promising indicator of peripheral perfusion; however, fluctuations in values owing to changes in the finger on which the probe is attached reduce its reliability. In this study, interfinger differences in PAI were examined, and the index and ring fingers showed the least difference. When using PPI as an indicator of peripheral perfusion in clinical practice or research, consistent values may be obtained by alternately attaching it to the index and ring fingers.

Two previous studies have reported that the interfinger differences in PPI are the least between the middle and ring fingers [14] or between the ring and little fingers [15], which differ from the results of the present study. There are two possible limitations to these previous studies. First, the PPI for each finger was measured sequentially using a single probe in both studies [14,15]; therefore, the PPI values at the same time point were not obtained. Second, because a clip-type probe was used in one study [15] (the other study did not mention which type of probe was used [14]), the pressure applied to the measurement site [23] may have differed due to differences in finger thickness, which may have affected the PPI value [20]. In the present study, the five fingers were measured simultaneously using tape-type probes; therefore, interfinger differences may have been more accurately verified.

The reason for the difference in PPI values between the fingers has not yet been clarified. Fingertip thickness may be a contributing factor to interfinger differences in PPI. PPI, such as PAI, is calculated as the ratio of the pulsatile component to the nonpulsatile component of the transmitted infrared light intensity [11]. The pulsatile component factor is arterial pulsation, and the nonpulsatile component factors are venous blood, bone, muscles, and connective tissue [11]. Because fingertip thickness corresponds to the bone, muscle, and connective tissues that constitute the non-pulsatile component, it was hypothesized that fingertip thickness could influence the calculation of PPI. However, in our study, no correlation was found between fingertip thickness and PAI (r = 0.06, *p* = 0.49). Factors other than fingertip blood flow, such as capillary density and autonomic regulation, may also contribute to interfinger differences in the PAI. Further research is required to clarify the interactions between these factors.

In our study, a strong positive correlation was observed between the mean PAI and the difference between the maximum and minimum PAI. As expected, a higher PAI was associated with a greater interfinger difference, whereas a lower PAI was associated with a smaller difference. This observed relationship implies that even if interfinger difference appears minimal during peripheral hypoperfusion, it may increase as peripheral perfusion improves. To ensure consistent and reliable assessment in such situations, probe repositioning should preferably be performed between the index and ring fingers, which demonstrated the least variability.

PPI, as measured using a pulse oximeter, is a potent indicator that can noninvasively, continuously, and quantitatively assess peripheral perfusion. Attempts have been made to calculate cutoff values for diagnosing peripheral hypoperfusion [10] and predicting mortality in critically ill patients [24-26], but these have not been definitive. To obtain accurate values, the interfinger differences in PPI should be considered. Based on the results of this study, the index and ring fingers have the smallest interfinger differences, and the use of these two fingers as measurement fingers when PPI is used in research and clinical practice is recommended.

The present study has a few limitations. First, this was a single-center exploratory study with a small sample size. While simultaneous PPI measurement across all fingers using tape-type probes offers advantages, further research with larger cohorts is needed to strengthen the study findings. Second, the study was conducted on healthy adults who may not represent critically ill patients with peripheral hypoperfusion, such as those with septic shock. This study might not have captured all physiological changes that occur in the peripheral perfusion of critically ill patients, such as vascular endothelial injury [27]. However, it is unlikely that these physiological changes would occur heterogeneously between fingers, which may not have had a significant impact on the results of this study. Finally, although tape-type probes were used in this study, clip-type probes have been used in both clinical and research settings. Whether the results of this study can be applied to clip-type probes is a matter of concern. For the clip type, the pressure produced by the spring tension may vary with the thickness of each finger [20]. As shown in the results, the index and ring fingers had the least interfinger differences, not only in PPI values but also in finger thickness; therefore, it is highly possible that the same results would be obtained with clip-type probes. Additionally, the formula for calculating PPI differs slightly depending on the device manufacturer, and the PPI value may differ depending on the measurement device [9]. However, as this study investigated the interfinger difference in PPI, we believe that the difference in measurement devices is unlikely to have affected the results.

## Conclusions

In this study, the PPI values measured using the pulse oximeter showed the smallest interfinger differences between the index and ring fingers. If it is necessary to change the finger to which the probe is attached during clinical or research use, it may be possible to consistently measure the PPI values by alternately attaching the probe to the index and ring fingers. However, further research is needed to strengthen these findings.
